# Exploring the Impact of Nanotherapeutics on Histone H3 and H4 Acetylation Enrichment in Cancer Epigenome: A Systematic Scoping Synthesis

**DOI:** 10.3390/epigenomes9040044

**Published:** 2025-11-07

**Authors:** Milad Shirvaliloo, Sepideh Khoee, Samideh Khoei, Roghayeh Sheervalilou, Parisa Mohammad Hosseini, Reza Afzalipour, Sakine Shirvalilou

**Affiliations:** 1 Finetech in Medicine Research Center, Iran University of Medical Sciences, Tehran 1449614525, Iran; shirvaliloo@tbzmed.ac.ir; 2 Department of Polymer Chemistry, School of Chemistry, College of Science, University of Tehran, Tehran 141556455, Iran; khoee@ut.ac.ir; 3 Department of Medical Physics, School of Medicine, Iran University of Medical Sciences, Tehran 1449614525, Iran; parisa.mhosseini1998@gmail.com; 4 Children and Adolescents Health Research Center, Research Institute of Cellular and Molecular Sciences in Infectious Diseases, Zahedan University of Medical Sciences, Zahedan 9816743463, Iran; sheervalilour@zaums.ac.ir; 5 Pharmacology Research Center, Zahedan University of Medical Sciences, Zahedan 9816743463, Iran; 6 Student Research Committee, Iran University of Medical Sciences, Tehran 1449614525, Iran; 7 Molecular Medicine Research Center, Hormozgan Health Institute, Hormozgan University of Medical Sciences, Bandar Abbas 7919693116, Iran; reza.afzalipoor@gmail.com; 8 Department of Radiology, Faculty of Para-Medicine, Hormozgan University of Medical Sciences, Bandar Abbas 7919693116, Iran

**Keywords:** nanotherapeutics, nanomaterials, cancer, histone acetylation, epigenetics, differential gene expression

## Abstract

**Background/Objectives**: Histone acetylation regulates gene expression and plays a key role in cancer pathophysiology. Nanotherapeutics are known to modulate histone acetylation and influence cancer progression. This systematic scoping review examines the effects of nanotherapeutics on histone acetylation enrichment across multiple cancers. **Methods**: A systematic search of Embase, PubMed/MEDLINE, Scopus, and Web of Science was conducted in accordance with the PRISMA 2020 statement. A total of 13 studies were included. Data were analyzed and visualized in R, and risk of bias was assessed with ToxRTool (OSF Registration: 10.17605/OSF.IO/E643S). **Results**: Nanotherapeutics were most commonly evaluated against breast (21.4%), prostate (21.4%), pancreatic (14.3%), and bladder (14.3%) cancers. Primary nanomaterials used in the synthesis of nanotherapeutics included poly(lactic-co-glycolic acid) (25.0%), gold (21.4%) and arsenic oxide (21.4%) nanoparticles. Studied histone acetylation marks included H3K9ac, H3K14ac, H3K27ac and H4K16ac. Treatment with nanotherapeutics increased histone H3 and H4 acetylation enrichment, particularly H3K14ac in colorectal and prostate cancers and H4K16ac in ovarian cancer. Conversely, gold-based nanotherapeutics decreased H3K9ac and H3K14ac enrichment in breast cancer. The optimal concentration for most nanotherapeutics was ≤25 µM, with PpIX-FFYSV showing the strongest anticancer effect (viability <25%). Across four preclinical studies (*n* = 58), treatment with the nanotherapeutics reduced tumor size to less than 50% of control in 64% of animals (95% CI: 21–92%, I^2^ = 63.8%). Altered histone acetylation was associated with differential expression of *CDKN1A*, *HSPA1*, *SREBF2* and *TGFB*. **Conclusions**: The evidence demonstrates that nanotherapeutics can alter histone acetylation patterns by modulating EP300/CBP, GCN5 and HDAC, preventing cancer progression and invasion.

## 1. Introduction

The development of nanotherapeutics has significantly advanced cancer therapy by overcoming key limitations of conventional treatments [1], including poor solubility, non-specific toxicity, and inadequate tumor targeting [2,3,4,5]. Established clinical applications include lipid-based systems such as polyethylene glycol (PEG)ylated liposomal doxorubicin [6,7,8], while polymeric nanoparticles like poly(lactic-co-glycolic acid) or PLGA have demonstrated utility in preclinical and early clinical studies for controlled drug delivery [9,10,11,12,13]. Research continues to expand into diverse nanomaterial platforms including metallic nanostructures such as gold [14,15,16] and silver nanoparticles for photothermal therapy [17,18,19], carbon-based carriers such as graphene oxide for combined chemo-photothermal approaches [20,21,22,23], protein-derived systems involving bovine serum albumin nanoparticles for drug encapsulation [24,25,26], and small molecule-based architectures encompassing peptide-functionalized dendrimers [27,28,29,30]. Additionally, biomimetic strategies, such as nanoparticles coated with macrophage membranes to enhance tumor homing, represent emerging experimental approaches [31,32,33]. The precision and cellular interaction capabilities of these nanotherapeutics enable not only targeted cytotoxicity [34,35] but also the modulation of epigenetic processes [36,37,38], with evidence indicating that specific nanotherapeutics can alter histone acetylation patterns in cancer cells [39,40,41]. This epigenetic dimension is a critical, yet underexplored, aspect of their anticancer mechanism [42].

Histone acetylation is vital for regulating gene expression through its impact on chromatin structure [43]. This process, facilitated by histone acetyltransferases (HATs) such GNAT, MYST, and EP300/CBP families, involves adding acetyl groups to lysine residues on histone tails [43,44]. This modification neutralizes the positive charge of lysines, resulting in uncondensed chromatin structure that enhances transcription [45]. Notably, acetylation of lysine 16 on histone H4 (H4K16ac) and lysine 27 on histone H3 (H3K27ac) are critical for gene regulation and implicated in cancer pathophysiology [46,47,48]. H4K16ac is actively involved reducing chromatin compaction, which is crucial for the maintenance of cellular functions [46]. Decreased levels of H4K16ac are associated with various cancers and may serve as a prognostic marker [49,50]. Additionally, H3K27ac is enriched at active promoters and enhancers, and counteracts gene silencing by preserving polycomb protein recruitment [51]. Dysregulation of H3K27ac can contribute to tumorigenesis by disrupting the expression of oncogenes and tumor suppressor genes [52,53,54]. Histone deacetylases (HDACs) counterbalance the action of HATs by removing acetyl groups, leading to chromatin condensation [55]. In cancer, overactive HDACs often result in poor outcomes by disturbing the acetylation equilibrium, which can subsequently activate oncogenes or silence tumor suppressors [56,57]. As such, targeting histone deacetylation presents a promising approach for cancer therapy [58]. HDAC inhibitors (HDACi) like vorinostat [59,60], belinostat [61,62], entinostat [63,64] and romidepsin [65,66] have been approved for the treatment of certain cancers. These agents function by reactivating tumor suppressors, or inhibiting oncogenes [58]. In recent years, nanoencapsulated formulations of these HDACi agents have been tested on experimental models of cancer [67,68,69].

Different nanoparticles (NPs) have been shown to significantly alter histone acetylation patterns across diverse cell types, suggesting epigenetic mechanisms as key contributors to nanotoxicity. For instance, exposure to iron-based magnetic NPs (Fe_2_O_3_ and Fe_2_O_3_@Co) in NIH3T3 fibroblast cells primarily induced changes in the genomic distribution of H3K27ac, which directly correlated with altered gene expression programs underlying toxicity [70]. Similarly, titanium dioxide (TiO_2_) NPs exposure in human colorectal (Caco-2) and lung (NL20) epithelial cells led to significant changes in multiple histone modifications, including reduced acetylation marks such as H3K9ac and H4K8ac in Caco-2 cells, and H3K9ac and H3K18ac in NL20 cells [2]. This disruption in histone acetylation was linked to aberrant expression of histone-modifying enzymes like HDAC9 and HAT1 [39]. Furthermore, amino-modified polystyrene NPs caused a global reduction in H3K9ac levels in both human lung epithelial and brain endothelial cells, an effect driven by nanoparticle-induced oxidative stress and associated with transcriptional repression and apoptosis [71]. While these studies collectively demonstrate that diverse NPs can disrupt histone acetylation profiles in different cell lines, often through oxidative stress pathways, the potential therapeutic application of this knowledge remains largely unexplored. Specifically, the capacity of nanotherapeutics to deliberately modulate histone acetylation patterns—thereby regulating genes involved in cancer pathogenesis—has rarely been systematically addressed, despite the clear evidence that nanotherapeutics can influence these critical epigenetic marks.

Considering the present knowledge gap in our understanding of the effects of nanotherapeutics on posttranslational histone acetylation patterns in cancer, the aim of this systematic scoping review is to synthesize evidence on potential impact of these nanotherapeutics on histone acetylation enrichment in tumor cells, with particular focus on the physicochemical properties of nanotherapeutics, as well as potential relationships between altered histone acetylation and differential gene expression and their combined effect on cancer cell viability.

## 2. Results

### 2.1. Systematic Search

**Figure 1:**
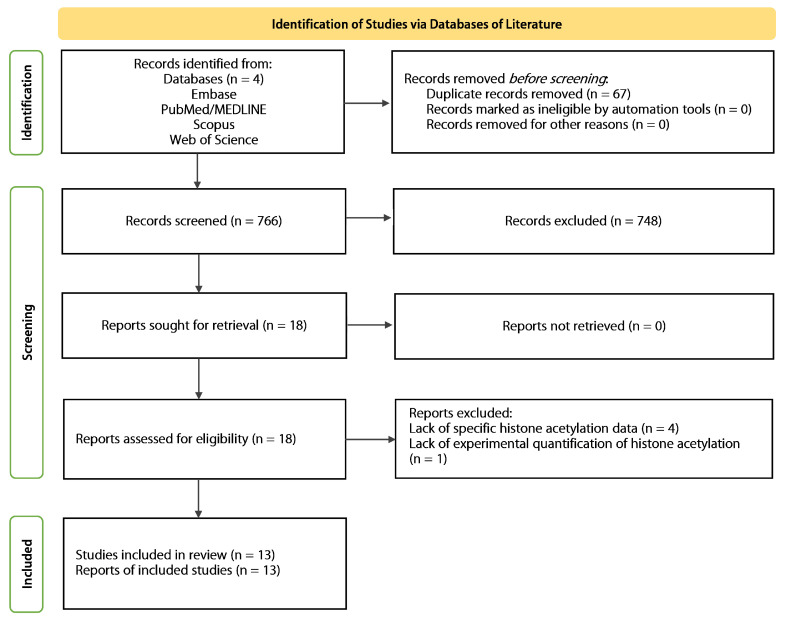
PRISMA flowchart of study identification and selection.

A comprehensive literature search across four databases—Embase, PubMed/MEDLINE, Scopus, and Web of Science—identified 833 records. After duplicate removal, 766 unique records were screened. After applying eligibility criteria, 13 studies were selected for evidence synthesis. The PRISMA flowchart of study identification and selection is shown in Figure 1.

### 2.2. Summary of the Included Studies

The 13 included studies provided 28 experimental entries. An experimental entry refers to a specific nanotherapeutics tested on a specific cancer model to measure acetylation levels of a specific histone mark. Table 1 summarizes the basic characteristics of the included studies, including demographic information, study design, and cancer models.

**Figure 2:**
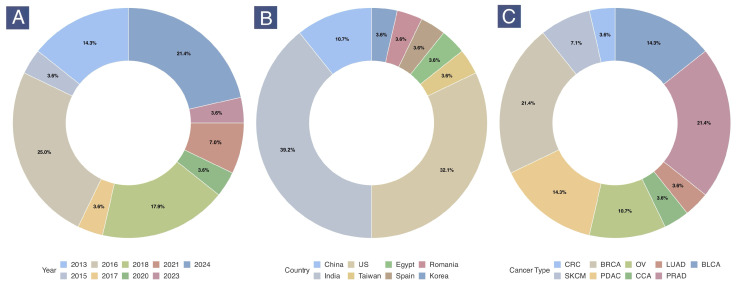
Frequency of publication year (**A**), country of origin (**B**), and cancer types (**C**) among the included studies. Note: All plots were generated based on the count of experimental entries (N = 28). (BLCA: bladder cancer; BRCA: breast cancer; CCA: cholangiocarcinoma; CRC: colorectal cancer; LUAD: lung cancer; OV: ovarian cancer; PDAC: pancreatic ductal adenocarcinoma; PRAD: prostate adenocarcinoma; SKCM: skin cutaneous melanoma.

The publication year, country of origin, and cancer type of the included studies are shown in Figure 2. Most experiments with these nanotherapeutics were conducted in 2016, 2018, and 2024 that primarily originated from India (39.3%), followed by the US (32.1%) and China (10.7%). The studies predominantly examined breast (21.4%), prostate (21.4%), bladder (14.3%), and pancreatic (14.3%) cancers.

### 2.3. Characteristics of Nanoformulations

The physicochemical characteristics of the included nanotherapeutics are summarized in Table 2.

**Figure 3:**
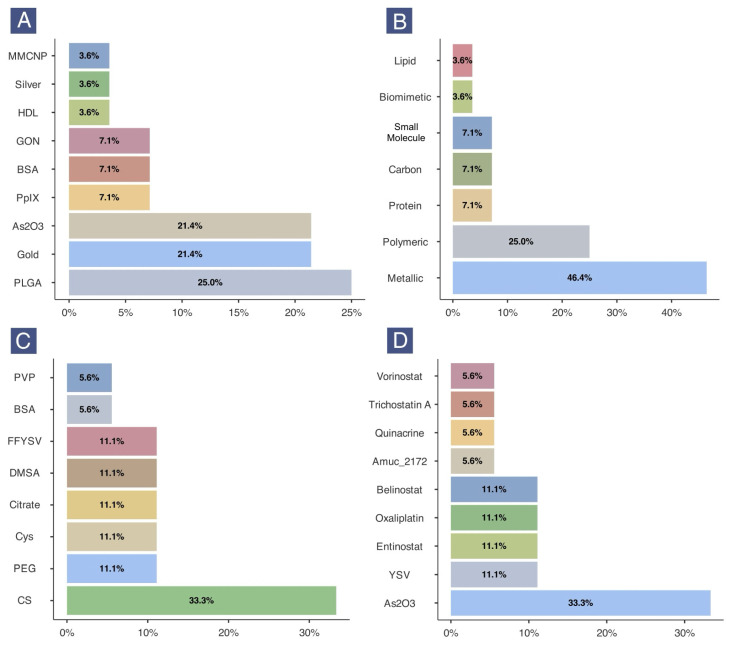
Frequency of nanomaterial type (**A**), nanomaterial class (**B**), surface functionalization (**C**), and anticancer agent (**D**) in the included nanotherapeutics. Note: All plots were generated based on the count of experimental entries (N = 28). (BAS: bovine serum albumin; CS: chitosan; Cys: cysteamine; DMSA: dimercaptosuccinic acid; FFYSV: L-phenylalanine-L-phenylalanine-tyroservatide; GON: graphene oxide nanosheet; HDL: high-density lipoprotein; MMNCP: macrophage membrane-coated nanoparticle; PEG: polyethylene glycol; PLGA: poly(lactic-co-glycolic acid); PpIX: protoporphyrin IX; PVP: polyvinylpyrrolidone).

Poly(lactic-co-glycolic acid) (PLGA) (25.0%) was the most common primary nanomaterial, followed by gold (21.4%) and arsenic oxide (21.4%). Silver, high-density lipoprotein (HDL), and macrophage membrane-coated nanoparticles (MMCNPs) were less frequently used (all 3.6%) (Figure 3A). When classified by nanomaterial type, metallic nanomaterials (46.4%) including gold, silver, and arsenic oxide were the most common. This was followed by polymeric nanomaterials (25%), which included only PLGA-based formulations. Biomimetic and small molecule classes included MMCNPs and protoporphyrin IX (PpIX), respectively (Figure 3B).

For surface functionalization, chitosan (CS) was most frequently used (33.3%), primarily in arsenic oxide nanoparticle (AsNP) treatments. Other functionalization materials included polyethylene glycol (PEG), citrate, cysteamine, and dimercaptosuccinic acid (DMSA) (all 11.1%) (Figure 3C). Regarding anticancer agents, arsenic oxide was used in 33.3% of experimental entries, followed by tyroservatide (YSV), entinostat (ENT), oxaliplatin (OXP), and belinostat (Bel) (all 11.1%) (Figure 3D).

### 2.4. Histone Acetylation Enrichment

The effects of nanotherapeutic dosages on histone acetylation enrichment and cancer cell viability in vitro are presented in Table 3.

**Figure 4:**
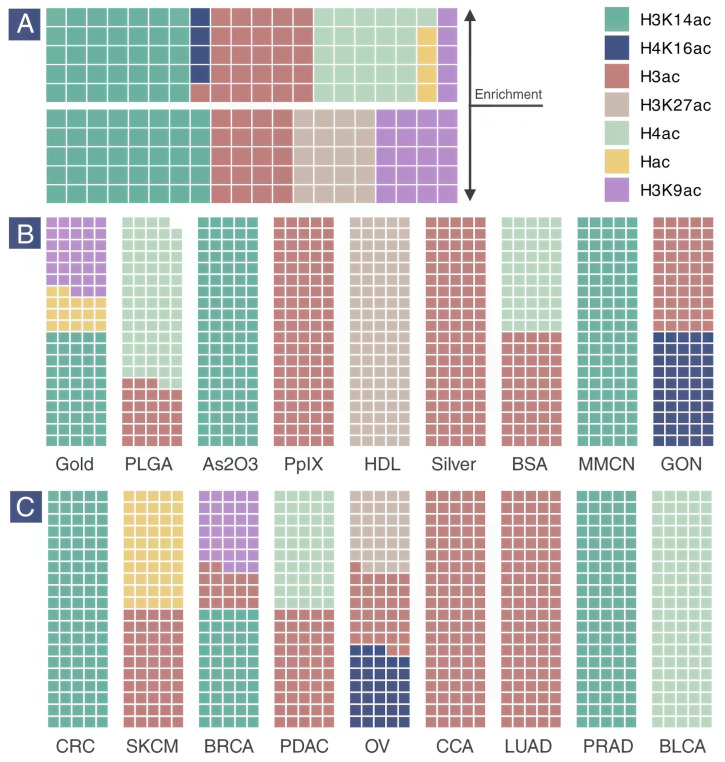
Distribution of histone acetylation marks based on enrichment pattern (**A**), nanomaterial type (**B**), and cancer type (**C**). Note: All plots were generated based on the count of experimental entries (N = 28). (BSA: bovine serum albumin; BLCA: bladder cancer; BRCA: breast cancer; CCA: cholangiocarcinoma; CRC: colorectal cancer; GON: graphene oxide nanosheet; HDL: high-density lipoprotein; LUAD: lung adenocarcinoma; MMCN: macrophage membrane-coated nanoparticle; OV: ovarian cancer; PDAC: pancreatic ductal adenocarcinoma; PLGA: poly(lactic-co-glycolic acid); PpIX: protoporphyrin IX; PRAD: prostate adenocarcinoma; SKCM: skin cutaneous melanoma).

Waffle plots were used to visualize the distribution of histone acetylation marks (Figure 4). Some studies did not specify the exact residue of histone acetylation; these were categorized as H3 acetylation (H3ac), H4 acetylation (H4ac), or simply histone acetylation (Hac). H3K14ac was the most frequently studied acetylation mark, followed by H3K9ac and H3K27ac. All marks are known to activate gene expression. H3 was the most commonly acetylated histone, with H3K14ac being the most prevalent type. H4 was less frequently studied than H3, but in all cases, acetylation of H4, including H4K16ac, was enriched without repression. All repression cases involved H3, with H3K9ac and H3K14ac being the dominant marks (Figure 4A).

The distribution of histone acetylation across different nanomaterials showed that H3 acetylation was most commonly affected by nanotherapeutics based on metals, proteins, and carbon. H3K9ac was only targeted by gold-based nanotherapeutics, while H4K16ac was exclusively targeted by graphene oxide nanosheets (GONs), and H3K27ac was studied only against HDL-like nanoparticles (Figure 4B). Histone acetylation was examined in 9 specific cancer types using different cell models. Ovarian and breast cancers were the most extensively studied types, with H3K27ac and H4K16ac (ovarian) and H3K9ac and H3K14ac (breast) being investigated. H4 acetylation was only studied in pancreatic and bladder cancers, suggesting specific relevance in these cancer types when evaluating responses to nanotherapeutics.

### 2.5. Nanotherapeutic Size, Dosage and Cancer Cell Viability

**Figure 5:**
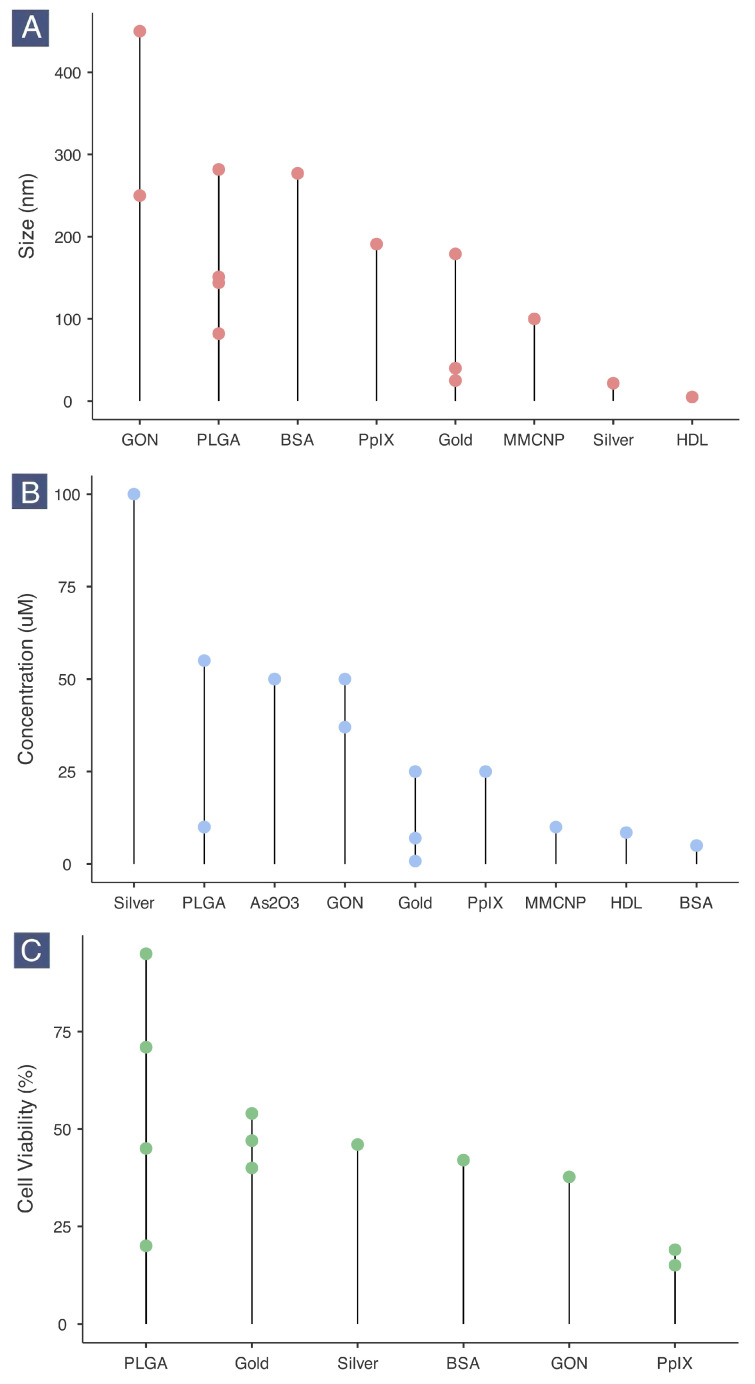
Lollipop plots of size (**A**), concentration (**B**), and cell viability (**C**) divided by nanomaterial classification. Note: Each dot represents a specific value reported for the variable. Plots are ordered to show efficiency in terms of size, dosage, and anticancer effect. (BSA: bovine serum albumin; GON: graphene oxide nanosheet; HDL: high-density lipoprotein; MMCNP: macrophage membrane-coated nanoparticle; PLGA: poly(lactic-co-glycolic acid); PpIX: protoporphyrin IX).

Nanotherapeutics ranged in size from 5 to approximately 400 nm. The smallest formulation was HDL-like nanoparticles (5 nm), followed by PVP-AgNPs (21.74 nm). Gold-based nanotherapeutics showed diverse sizes depending on surface functionalization and drug encapsulation, with the smallest at 25 nm. On the other end of the spectrum, GO-based formulations ranged from over 200 nm to approximately 400 nm due to the nanosheet structure of graphene oxide (Figure 5A).

The concentration of nanotherapeutics ranged from 0.8 to 100 µM, with quinacrine-loaded AuNPs and PVP-AgNPs tested at the lowest and highest concentrations, respectively (Figure 5B). Most studies did not report IC50 values, but the concentrations selected generally produced significant anticancer effects. The optimal concentration for BSA, HDL, MMCNP, PpIX, and gold-based nanotherapeutics was 25 µM or less, indicating efficient delivery and uptake by cancer cells. This finding aligns with the protein (BSA, PpIX), lipoprotein (HDL), and bilayer membranous (MMCNP) nature of these nanotherapeutics, which are highly biocompatible and efficiently taken up [85,86].

Specific percentages for cancer cell viability were reported in only a few studies, with heterogeneous values observed (Figure 5C). The least effective nanotherapeutic was unloaded PGON/PLGA-NPs, which did not affect cancer cell viability without a drug. When loaded with belinostat (Bel), these nanoparticles reduced cell viability to 20% at 10 µM concentration. Gold-based nanotherapeutics produced viability rates close to 50% at optimal concentrations. The most effective nanotherapeutic was PpIX-FFYSV, consistently reducing cell viability to below 25%. GON and BSA-based formulations resulted in viability rates between 40 and 50%, suggesting potential anticancer effects under controlled conditions.

### 2.6. Differential Gene Expression in Cancer Cells

Five of the 13 included studies reported differential gene expression following altered histone acetylation patterns after treatment with nanotherapeutics (Table 4). Enrichment of histone H3 acetylation (particularly H3K14ac) positively regulated HSPA1 [75] and CDKN1A [82,83], through GCN5 stimulation and HDAC inhibition, respectively. One study reported decreased H3K14ac enrichment due to reduced EP300 activity, accompanied by downregulation of TGFB in cancer cells [74]. SREBF2 was another gene downregulated as a result of reduced EP300 activity, associated with decreased H3K27ac enrichment [73].

### 2.7. Anticancer Effects of Nanotherapeutics in Animal Models of Cancer

In vivo experiments conducted in murine cancer models further supported the anticancer potential of various nanotherapeutics. PLGA-based and gold-based nanotherapeutics were among the most frequently employed and consistently demonstrated reductions in tumor size in various cancers, although changes in body weight were not always significant. HDL- and peptide-based nanotherapeutics also resulted in notable tumor suppression. Importantly, MMCNPs not only significantly diminished tumor count and growth, but also improved body weight in animals. However, marked heterogeneity was observed in the administered dosages and treatment durations, reflecting differences in nanomaterial composition and whether the formulations encapsulated an active anticancer agent. Collectively, treatment with the included nanotherapeutics universally resulted in tumor size reduction (Table 5).

**Figure 6:**
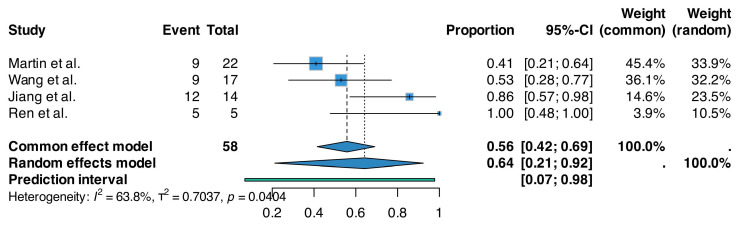
Forest plot of the pooled proportion of animals exhibiting tumor size reduction to less than 50% of that observed in control groups following treatment with nanotherapeutics based on data extracted from Wang et al. [73], Jiang et al. [75], Ren et al. [76] and Martin et al. [84]. The analysis was performed using a random-effects model with logit transformation of proportions. Horizontal lines represent 95% confidence intervals, and the diamond indicates the pooled estimate.

A random-effects meta-analysis of proportions demonstrated that treatment with the evaluated nanotherapeutics led to tumor size reduction to less than 50% of that observed in the corresponding control groups in 64% (95% CI: 21–92%, *p* = 0.040) of animals across the included studies with moderate to high heterogeneity (*n* = 58, I^2^ = 63.8%) (Figure 6).

### 2.8. Risk of Bias Assessment

**Figure 7:**
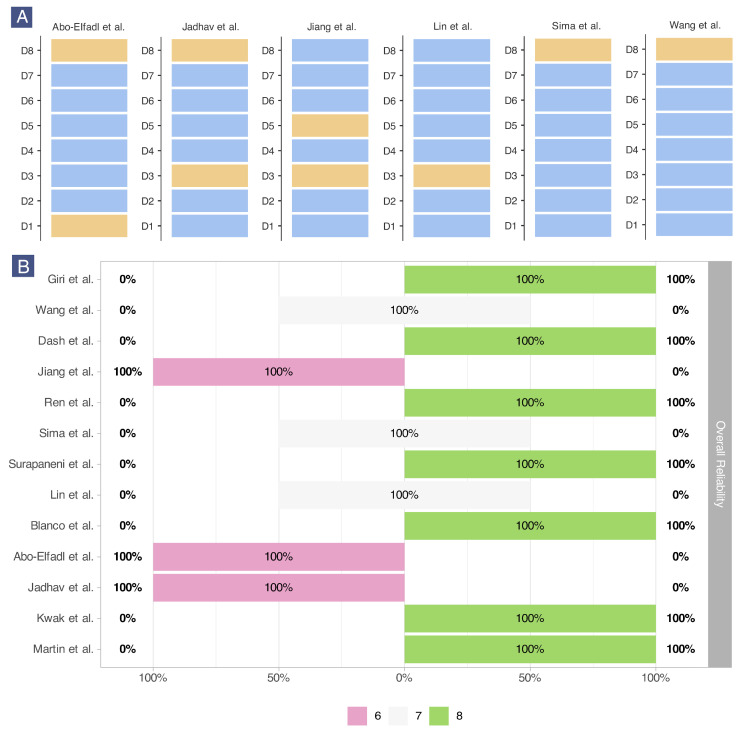
Risk of bias assessment of the included studies [72,73,74,75,76,77,78,79,80,81,82,83,84] based on ToxRTool. (**A**) Stacked boxplots of domains by study label, showing yes/no responses for each domain. Blue indicates “yes”, yellow indicates “no”. (**B**) Likert plot of reliability scores showing studies grouped by scores of 6, 7, or 8.

Risk of bias was assessed using ToxRTool. Figure 7 shows the summary of study reliability based on the 8 mandatory ToxRTool criteria. Full details of criterion eligibility for each study are provided in Table S1.

As shown in Figure 7A, 6 of the 13 studies (46.1%) did not meet all 8 criteria, resulting in scores below 8. The most frequently unmet domain was D8 (endpoint measures and analytical methods), which was not met by 4 studies. These studies often did not report specific values for cancer cell viability, apoptosis, or the exact histone acetylation residue. Domain D3 (test substance characterization) was not met by 3 studies, which frequently lacked specific physicochemical characteristics of their nanotherapeutics. Overall, 3 studies scored 6/8, 3 studies scored 7/8, and 7 studies scored 8/8 (Figure 7B).

The majority of studies (53.9%) were considered reliable without restrictions, while 46.1% were reliable with restrictions. No studies were deemed unreliable.

## 3. Discussion

This scoping review synthesizes evidence demonstrating that exposure to nanotherapeutics is often associated with increased histone acetylation in cancer cells (Table 3). Specifically, treatments with nanoparticle-based formulations induce enrichment of key acetylation marks, including H3K9ac, H3K14ac, H3K27ac, and H4K16ac, through mechanisms involving either the stimulation of acetyltransferase activity or the inhibition of deacetylase function. These epigenetic alterations correlate with reduced cancer cell viability by means of differential gene expression (Table 4), suggesting that modulation of histone acetylation represents a measurable mechanism contributing to the therapeutic efficacy of these nanotherapeutics.

H3K14ac is recognized as a functionally significant histone modification in cancer biology, with studies demonstrating its involvement in tumorigenesis and treatment response. In triple-negative breast cancer, H3K14ac distinguishes malignant cells from normal tissue [87]. In prostate cancer, the bromodomain of BAZ2A binds H3K14ac-enriched chromatin at inactive enhancers, repressing transcription of genes silenced in aggressive cancers. Disruption of this interaction may impair cancer stem cell properties [88]. Similarly, in kidney cancer, the tumor suppressor PBRM1 binds H3K14ac via its bromodomains, and mutations disrupting this interaction alter gene expression and compromise tumor suppression [89]. In non-small-cell lung cancer, the YEATS domain of GAS41 recognizes H3K14ac to facilitate histone H2A.Z deposition, supporting cancer cell proliferation and survival. Targeting this interaction inhibits growth in vitro and in vivo [90]. Additionally, ANCCA, an ATPase linked to tumorigenesis, interacts with H3K14ac to regulate cell cycle gene expression and proliferation [91].

H3K9ac regulates gene expression and exhibits context-dependent associations with cancer progression. In pancreatic cancer, H3K9ac represses cancer cell viability, colony formation, and migration via modulation of the Ras-ERK pathway. Downregulation of H3K9ac through MDM2-mediated PCAF degradation—driven by Ras ERK1/2 activation—promotes pancreatic carcinogenesis [92]. In breast cancer, elevated H3K9ac correlates with poorer prognosis in Her2-positive and Ki67-positive subtypes, suggesting potential utility as a prognostic marker [93]. Similarly, reduced H3K9ac expression in oral squamous cell carcinoma (OSCC) associates with increased cell proliferation, epithelial–mesenchymal transition (EMT), and worse clinical outcomes [94]. In contrast, gastric cancer studies report no significant difference in H3K9ac expression between tumor and normal tissues, with no correlation to clinicopathological features or survival [95]. In ovarian cancer, decreased H3K9ac correlates with higher malignancy, lower histological grading, and advanced clinical staging [96]. These observations indicate that H3K9ac’s role in cancer is not universally suppressive or promotive but varies by tumor type and molecular context.

Histone acetylation/deacetylation dynamics represent a key epigenetic mechanism in cancer, with dysregulation contributing to aberrant gene expression and tumor progression [97,98]. In this context, histone deacetylase inhibitors (HDACis) have emerged as promising therapeutic agents capable of reactivating silenced tumor suppressor genes and inducing apoptosis [99]. However, clinical translation of HDACis is limited by poor solubility, low bioavailability, and narrow therapeutic indices [100]. Nanomedicine offers a solution by enabling enhanced delivery and targeted delivery of HDACis. For example, encapsulation of vorinostat in biocompatible nanocarriers improves drug stability, bioavailability, and tumor accumulation, thereby augmenting therapeutic efficacy [101,102,103]. In this review, we specifically evaluated three distinct PLGA-encapsulated nanoformulations of entinostat, vorinostat and belinostat that were tested against pancreatic cancer [72], cholangiocarcinoma [83] and bladder cancer cells [84], respectively, demonstrating promising tumor growth inhibition compared to free drug administration. Multifunctional nanotherapeutics combining HDACis with agents such as metformin or triptolide further demonstrate synergistic suppression of EMT and metastasis [104]. Notably, nanostrategies addressing intracellular delivery challenges have proven effective; e.g., a dendritic nanohybrid formulation conjugating HDACis with quantum dots enhanced nuclear drug localization and potently inhibited lung cancer cell growth [105]. Similarly, nanocarriers co-delivering HDACis with radiosensitizers amplify DNA damage and overcome radioresistance during radiotherapy [106].

Based on the leveraged evidence, nanotherapeutic-mediated epigenetic reprogramming orchestrates a coordinated anticancer response through targeted regulation of key genes via histone acetylation dynamics. Consistent with evidence from studies evaluating diverse nanotherapeutics (metallic, polymeric, biomimetic, and lipid-based), these agents modulate specific histone marks to suppress oncogenic pathways while activating tumor-suppressive mechanisms. The downregulation of TGF-β—driven by reduced H3K14ac via EP300/CBP inhibition [74]—disrupts intratumoral immunosuppression earlier in cancer pathogenesis [107]. This suppression concurrently reverses EMT, inhibits invasion, and restores immune surveillance, effectively halting metastatic progression [108]. Parallel suppression of SREBF2, mediated by decreased H3K27ac through EP300/CBP downregulation [73], undermines cancer cell metabolic plasticity. As a master regulator of cholesterol biosynthesis, SREBF2 depletion decreases essential membrane components and signaling lipids, repressing tumor proliferation in nutrient-scarce microenvironments [109,110]. The dual targeting of TGF-β and SREBF2 creates a synergistic metabolic-invasive collapse that could starve tumors of structural resources and migratory capacity.

**Figure 8:**
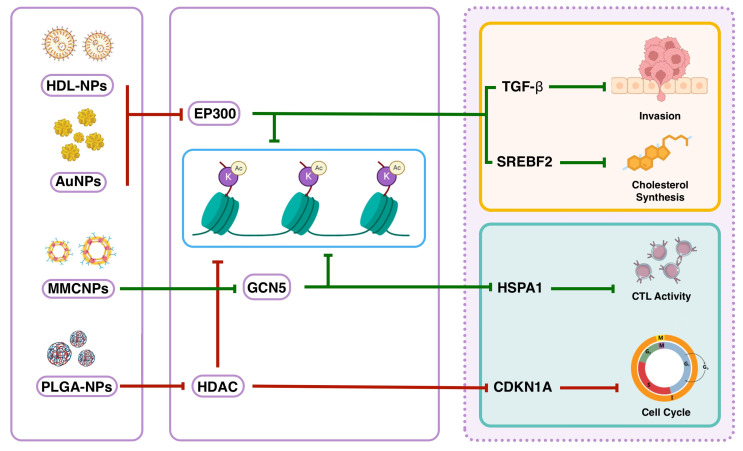
Mechanistic illustration of nanotherapeutic formulation-mediated epigenetic reprogramming in cancer cells. Nanotherapeutics modulate histone acetylation through EP300/CBP inhibition, HDAC blockade, and GCN5 activation to simultaneously downregulate oncogenic TGF-β/SREBF2 signaling and activate CDKN1A/HSPA1–dependent stress responses. This coordinated regulation leads to metabolic deprivation, loss of invasive potential, cell cycle arrest, and enhanced CTL-mediated apoptosis. Green arrows indicate activation; red arrows indicate inhibition. (AuNPs: gold nanoparticles; CTL: cytotoxic T lymphocyte; HDAC: histone deacetylase; HDL: high-density lipoprotein; MMCNP: macrophage membrane-coated nanoparticles; PLGA: poly(lactic-co-glycolic acid).

Concurrently, nanotherapeutics induce the upregulation of CDKN1A (p21) through two complementary pathways, i.e., direct H3K14ac reduction (via EP300/CBP inhibition) and HDAC inhibition-mediated H3 acetylation [82,83]. CDKN1A activation enforces G1/S cell cycle arrest, preventing DNA replication in stressed cells and sensitizing them to apoptosis [111,112]. This effect can potentially be amplified by HSPA1 hyperacetylation—driven by GCN5-mediated H3K14ac enhancement—which overwhelms proteostasis [113]. Despite its canonical pro-survival role, sustained HSPA1 overexpression enhances cytotoxic T lymphocyte (CTL)-mediated immune response, which has been confirmed in colorectal cancer cells exposed to MMNCPs carrying a GCN5 analogue [75]. Critically, CDKNA1-mediated cell cycle arrest and HSPA1-induced CTL response to bypass common resistance mechanisms through senescence or apoptosis [75,112]. This integrated mechanism—simultaneously targeting TGF-β/SREBF2 to disable invasion and metabolism, and HSPA1/CDKN1A to enforce cell death—explains the superior efficacy of nanotherapeutics. Figure 8 shows a simplified illustration of this putative mechanistic crosstalk.

By co-activating metabolic deprivation (via SREBF2 inhibition), loss of invasive potential (via TGF-β suppression), and stress/CTL-induced apoptosis (via HSPA1/CDKN1A), these agents generate a lethal epigenetic storm that suppresses tumor growth, metastasis, and therapy resistance. However, the current evidence is limited and further studies are required to confirm such speculations.

### Limitations

This systematic scoping review was constrained by the nascent state of research on nanotherapeutic-induced epigenetic modulation. Despite searching four major databases (Embase, PubMed/MEDLINE, Scopus, and Web of Science) without language or publication date restrictions, the limited number of included studies (*n* = 13) reflects the field’s underexplored nature. These studies exhibited substantial heterogeneity in experimental design, nanotherapeutic types, and acetylated histone lysine residues, precluding quantitative synthesis or statistical analysis. While categorical data were extracted and visualized to address this gap, the absence of standardized outcome measures and sufficient quantitative variables limited the depth of interpretation. Narrative synthesis, though appropriate for scoping reviews, inherently risks subjective interpretation; however, we mitigated this through transparent, evidence-based reporting of observed associations. Future systematic reviews with expanded datasets may enable more robust analytical approaches. Importantly, while more than half of the included studies were deemed reliable without restrictions, six of the thirteen studies were considered reliable with restrictions due to limitations in nanotherapeutic characterization and endpoint assessments of cancer cell viability and apoptosis. Nevertheless, all studies consistently demonstrated significant anticancer effects of the nanotherapeutics reviewed in this work.

## 4. Materials and Methods

### 4.1. Protocol and Registration

The review question was formulated using a slightly modified PEO (Population, Exposure, Outcome) framework. In this framework, the population consisted of experimental cancer models, the exposure was nanotherapeutics, and the outcome was alteration in histone acetylation enrichment. To identify relevant studies, we conducted a comprehensive search of systematic review databases, including the Cochrane Registry of Systematic Reviews. The review protocol was registered with the Open Science Framework (DOI: 10.17605/OSF.IO/E643S).

### 4.2. Eligibility Criteria

Studies were included if they: (1) reported original findings on the experimental application of nanotherapeutics in cancer, (2) used appropriate in vitro, in vivo, or human models, (3) characterized the physicochemical properties of the nanotherapeutics, and (4) evaluated the impact of these nanotherapeutics on histone acetylation enrichment in cancer models.

Exclusion criteria included (1) studies that did not explicitly report alterations in histone acetylation enrichment, (2) only mentioned anticancer agents capable of altering histone modifying enzymes without specifying histone acetylation measurements, or (3) lacked sufficient methodological details about the cancer models where histone acetylation status was determined. Review articles, editorial materials, commentaries, perspectives, and abstracts were also excluded.

### 4.3. Information Sources and Search

We searched four major databases including Embase, PubMed/MEDLINE, Scopus, and Web of Science Core Collection up to November 2025 in accordance with the PRISMA 2020 statement guidelines [114]. Search queries were developed using synonyms or equivalents for three primary keywords: (1) cancer, (2) histone acetylation, and (3) nanotherapeutic. In addition to general synonyms for histone acetylation, specific marks including H3K4ac, H3K9ac, H3K14ac, H3K18ac, H3K23ac, H3K27ac, H3K36ac, H3K56ac, H3K79ac, H3K122ac, H4K5ac, H4K8ac, H4K12ac, H4K16, H4K20ac and H4K91ac were included in the queries. The complete list of these synonyms and equivalents appears in Supplementary Table S2.

For PubMed/MEDLINE, specific MeSH terms were used. For Embase, synonyms were retrieved from Emtree and used to formulate the queries, with the ‘/exp’ operator applied to broaden the search scope. The search was conducted in title, abstract, and keyword fields across all databases without restrictions on cancer type, language, or publication date. Detailed search queries for all databases are provided in Supplementary Table S3.

### 4.4. Selection of Sources of Evidence

Identified records from the literature databases were uploaded to Rayyan “https://www.rayyan.ai/ (accessed on 1 March 2024)”. After duplicate removal (*n* = 67), titles and abstracts of the remaining records (*n* = 766) were independently screened by two reviewers (M.S. and R.S.). Potentially eligible studies were marked for full-text retrieval and evaluated in a second screening stage using the same approach. Inter-reviewer agreement during title/abstract and full-text screening was assessed using Cohen’s kappa taking into account disagreement with regard to one study that was ultimately deemed as ineligible. The calculated Cohen’s kappa indices for the two screening stages were κ ≈ 0.84 and κ ≈ 0.85, respectively, indicating substantial consistency between reviewers. Discrepancies were resolved through discussion with a third reviewer (R.A.) and consensus. Study selection followed the Joanna Briggs Institute (JBI) methodological guidelines [115], and eligible studies were included for evidence synthesis. The results of the systematic search and study identification were reported in accordance with the PRISMA 2020 reporting guidelines [114].

### 4.5. Data Extraction

Data extraction was conducted by two reviewers using a predefined data extraction form. The form included categorical, ordinal, and continuous variables: study ID, publication date, study design, cancer model, cancer type, nanoformulation, nanomaterial type and size, drug encapsulation status, anticancer agent, nanoformulation concentration, cancer cell viability, histone acetylation mark, and enrichment status. A third reviewer verified the extracted data and resolved discrepancies. The extracted data sheet is provided as Supplementary File S1.xlsx.

### 4.6. Critical Appraisal of Sources of Evidence

The quality of included studies was assessed using ToxRTool, developed by the Joint Research Center of the European Commission. This tool is recommended for evaluating in vitro studies of drug exposure [116]. ToxRTool assesses eight mandatory domains, each answered with yes or no: (1) test system identification and characterization; (2) test system maintenance and culture conditions; (3) test substance identification and characterization; (4) vehicle/solvent and its controls; (5) dose selection and concentrations; (6) exposure conditions; (7) positive/negative controls; and (8) endpoint measurement and analytical methods. Studies fulfilling all eight domains (8/8) were considered reliable without restrictions. Studies fulfilling six or seven domains (6/8 or 7/8) were considered reliable with restrictions. Studies with fewer than six positive responses were deemed unreliable [117]. Assessment results were visualized as individual box plots for each study and cumulative Likert plots using the ggstatsplot package “https://indrajeetpatil.github.io/ggstatsplot/ (accessed on 1 April 2025)” in R [118,119].

### 4.7. Synthesis of Evidence

Due to heterogeneity in methodologies and outcomes among the included studies, a narrative synthesis was conducted. The synthesis focused on nanoformulation characteristics, anticancer effects, histone acetylation outcomes, and differential gene expression when reported by studies. Findings were either visualized as distribution plots or presented qualitatively. To enhance clarity, extracted data were visualized using the ggstatsplot package in R. Charts displayed frequencies (percentages) for categorical variables and value distributions (ranges) for continuous variables. Given the diversity of study designs (various in vitro cancer models and nanomaterial types) and limited numerical data regarding anticancer outcomes, only meta-analysis of proportions was feasible for tumor size data of animal models. This was conducted in RStudio 2025.05.1+513 using metafor package and random-effects model. The PRISMA-ScR checklist detailing the methodology of this work is provided as Supplementary Checklist S1.

## 5. Conclusions

The present systematic scoping synthesis demonstrates that nanotherapeutics can influence histone acetylation patterns in cancer models, with H3K14ac being the most commonly examined mark. Various nanotherapeutic types, including PLGA-NPs, AuNPs, and arsenic oxide, showed differential effects on specific histone acetylation marks across different cancer types. Nanotherapeutics generally increased histone acetylation, with concentrations ≤25 µM demonstrating significant anticancer effects in most cases. The association between histone acetylation alterations and differential gene expression was documented in five studies, suggesting potential mechanisms for the observed anticancer effects. The findings indicate that nanotherapeutics may serve as promising tools for modulating epigenetic patterns in cancer, with specific formulations showing selective targeting of particular histone marks.

This review highlights key design principles for translating nano-epigenetic therapies into clinical practice. The consistent association between histone acetylation changes—particularly enrichment of H3ac, H4ac, and specific lysine marks—and reduced tumor viability across diverse in vitro models and multiple cancer types in vivo underscores the therapeutic relevance of targeting epigenetic regulation. Critically, the efficacy of nanotherapeutics without traditional chemotherapeutics (e.g., certain AuNPs, AgNPs) suggests that nanomaterials themselves can exert epigenetic effects, informing future designs that integrate carrier and active functions. For clinical translation, standardized assessment of toxicity profiles, scalable manufacturing of stable formulations with defined physicochemical properties, including but not limited to size < 500 nm and appropriate surface functionalization, and rigorous in vivo validation in immunocompetent models are essential next steps. These findings provide a practical framework for developing nano-epigenetic agents with enhanced specificity and reduced off-target effects in future anticancer strategies.

While further standardized studies are needed to establish clearer patterns and mechanisms, this systematic scoping review provides valuable insights into the relationship between nanotherapeutics and histone acetylation in cancer models. As the first review addressing this specific topic, it lays a foundation for future research on epigenetic modulation through nanotherapeutics in cancer therapy.

## Figures and Tables

**Table 1:** Summary of the included studies.

Study	Year	Country	Design	Experimental Entries	Cancer		Ref.
					Type *	Cell Model	
Giri et al.	2024	US	In vitro	4	PDAC	PANC-1 KPC	[72]
Wang et al.	2024	US	In vitro In vivo	1	OV	OVCAR5	[73]
Dash et al.	2024	India	In vitro In vivo	1	BRCA	MCF-7 T47D	[74]
Jiang et al.	2023	China	In vitro In vivo	1	CRC	CT26	[75]
Ren et al.	2021	China	In vitro In vivo	2	BRCA	4T1	[76]
					OV	A2780/Taxol	
Sima et al.	2020	Romania	In vitro	1	SKCM	SKmel23	[77]
Surapaneni et al.	2018	India	In vitro	4	BRCA	MDA-MB-231	[78]
Lin et al.	2018	Taiwan	In vitro	1	OV	SK-OV-3	[79]
Blanco et al.	2017	Spain	In vitro	1	LUAD	A549	[80]
Abo-Elfadl et al.	2016	Egypt	In vitro In vivo	1	SKCM	Skmel28	[81]
Jadhav et al.	2016	India	In vitro	6	PRAD (AR^+^)	LNCaP	[82]
					PRAD (AR^−^)	PC-3	
Kwak et al.	2015	Korea	In vitro In vivo	1	CCA	HuCC-T1	[83]
Martin et al.	2013	US	In vitro In vivo	4	BLCA (RB1^wt^)	T-24	[84]
					BLCA (RB1^mut^)	UM-UC-3	

AR: androgen receptor; BLCA: bladder cancer; BRCA: breast cancer; CCA: cholangiocarcinoma; CRC: colorectal cancer; LUAD: lung adenocarcinoma; OV: ovarian cancer; PDAC: pancreatic ductal adenocarcinoma; PRAD: prostate adenocarcinoma; RB1^mut^: RB1 mutated; RB1^wt^: RB1 wild-type; SKCM: skin cutaneous melanoma. * Abbreviations are based on The Cancer Genome Atlas designations.

**Table 2:** Physicochemical characteristics of nanotherapeutics in the included studies.

Study	Nanotherapeutic	Nanomaterial	Surface Functionalization	Size (nm)	Drug Encapsulation	Anticancer Agent	Ref.
Giri et al.	OXP@BSA-NPs	BSA	None	277.1	Yes	Oxaliplatin	[72]
	ENT@PLGA-NPs	PLGA		281.8		Entinostat	
Wang et al.	HDL-NPs	HDL	None	5.0	No	HDL-NPs + CDDP *	[73]
Dash et al.	QAuNPs	Gold	None	179.0	Yes	Quinacrine	[74]
Jiang et al.	Amuc_2172@MMCNPs	MMCNP	None	≈100.0	Yes	Amuc_2172	[75]
Ren et al.	PpIX-FFYSV	PpIX	FF-YSV	191.0	No	PpIX	[76]
Sima et al.	TSA@BSA-GONs	GON	BSA	250.0	Yes	Trichostatin A	[77]
Surapaneni et al.	Cit-AuNPs	Gold	Citrate	≈40.0	No	AuNPs	[78]
	Cys-AuNPs		Cysteamine	≈25.0			
Lin et al.	GONs	GON	None	450.0	No	GONs + CDDP *	[79]
Blanco et al.	PVP-AgNPs	Silver	PVP	21.74	No	AgNPs	[80]
Abo-Elfadl et al.	PEG-AuNPs	Gold	PEG	25.0	No	AuNPs	[81]
Jadhav et al.	AsNPs	As_2_O_3_	None	−	No	As_2_O_3_	[82]
	CS-AsNPs		Chitosan				
	DMSA-AsNPs		DMSA				
Kwak et al.	rINN@PEG/PLGA-NPs	PLGA	PEG	82.12	Yes	Vorinostat	[83]
Martin et al.	Bel@PGON/PLGA-NPs	PLGA	PGON Avidin	151.0	Yes	Belinostat	[84]
	PGON/PLGA-NPs			144.0		−	

As: arsenic; BSA: bovine serum albumin; CDDP: cisplatin; CS: chitosan; DMSA: dimercaptosuccinic acid; ENT: entinostat; FF: L-phenylalanine-L-phenylalanine; GON: graphene oxide nanosheet; HDL: high-density lipoprotein; MMCNP: macrophage membrane-coated nanoparticle; OXP: oxaliplatin; PEG: polyethylene glycol; PGON: poly(guanidinium oxanorbornene); PLGA: poly(lactic-co-glycolic acid); PpIX: protoporphyrin IX; PVP: polyvinylpyrrolidone; rINN: vorinostat; TSA: trichostatin A; YSV: tyroservatide. * CDDP was used as combination treatment.

**Table 3:** Overview of nanotherapeutic concentrations, associated histone acetylation enrichment patterns, and resultant effects on cancer cell viability in vitro.

Study	Treatment		Therapeutic Outcome				Ref.
	Nanotherapeutic	Optimal Concentration (µM)	Histone Modification		Antitumor Effect		
			Acetylation	Enrichment *	Viability (%)	Apoptosis (%)	
Giri et al.	ENT@PLGA-NPs	10	H3ac	↑	71	18.1	[72]
	OXP@BSA-NPs	5	H4ac	↑	42	25.4	
Wang et al.	HDL-NPs	8.5	H3K27ac	↓	↓	↑	[73]
Dash et al.	QAuNPs	0.8	H3K14ac	↓	≈47	↑	[74]
Jiang et al.	Amuc_2172@MMCNPs	10	H3K14ac	↑	↓	↑	[75]
Ren et al.	PpIX-FFYSV	25	H3ac	↑	≈35–48	↑	[76]
Sima et al.	TSA@BSA-GONs	37	H3ac	↑	↓	↑	[77]
Surapaneni et al.	Cys-AuNPs	25	H3K9ac	↑	≈47	≈18	[78]
	Cit-AuNPs	25		↓	≈54	≈13	
	Cys-AuNPs	25	H3K14ac	↑	≈47	≈18	
	Cit-AuNPs	25		↓	≈54	≈13	
Lin et al.	GONs (+CDDP)	50 (+200)	H4K16ac	↑	37.7	17.0	[79]
Blanco et al.	AgNPs	100	H3ac	↓	≈46	14.5	[80]
Abo-Elfadl et al.	PEG-AuNPs	7.0	Hac	↑	≈40	↑	[81]
Jadhav et al.	AsNPs	50	H3K14ac	↑	↓	↑	[82]
	CS-AsNPs	50					
	DMSA-AsNPs	50					
Kwak et al.	rINN@PEG/PLGA-NPs	55	H3ac	↑	≈45	↑	[83]
Martin et al.	Bel@PGON/PLGA-NPs	10	H4ac	↑	≈20	↑	[84]
	PGON/PLGA-NPs	10			≈95	Negligible	

* ↑ and ↓ indicate upregulation and downregulation, respectively. CDDP: cisplatin; CS: chitosan; DMSA: dimercaptosuccinic acid; ENT: entinostat; FF: L-phenylalanine-L-phenylalanine; GON: graphene oxide nanosheet; MMCNP: macrophage membrane-coated nanoparticle; OXP: oxaliplatin; PEG: polyethylene glycol; PGON: poly(guanidinium oxanorbornene); PLGA: poly(lactic-co-glycolic acid); PpIX: protoporphyrin IX; rINN: vorinostat; TSA: trichostatin A; YSV: tyroservatide.

**Table 4:** Differential gene expression and associated histone acetylation changes in cancer cells following in vitro treatment with nanotherapeutics.

Study	Anticancer Agent	Histone Modifier		Histone Enrichment		Differential Gene Expression		Ref.
		Enzyme Class	Activity *	Mark	Enrichment	Gene	Expression	
Wang et al.	CDDP	EP300/CBP	↓	H3K27ac	↓	SREBF2	↓	[73]
Dash et al.	Quinacrine	EP300/CBP	↓	H3K14ac	↓	TGFB	↓	[74]
Jiang et al.	Amuc_2172	GCN5	↑	H3K14ac	↑	HSPA1	↑	[75]
Jadhav et al.	As2O3	−	−	H3K14ac	↑	CDKN1A	↑	[82]
Kwak et al.	rINN	HDAC	↓	H3ac	↑	CDKN1A	↑	[83]

* ↑ and ↓ indicate upregulation and downregulation, respectively. CDDP: cisplatin; EP300/CBP: E1A binding protein p300/CREB binding protein; GCN5: general control non-derepressible 5; HDAC: histone deacetylase; rINN: vorinostat; TGFB: transforming growth factor beta.

**Table 5:** Summary of in vivo studies evaluating nanotherapeutic interventions in murine models of cancer, including dosage, treatment duration, route of administration, and anticancer outcomes.

Study	Nanotherapeutic	Cancer *		Treatment			Outcome ^†^			Ref.
							TC	TS	BW	
		Type	Animal Model	Dosage	Duration (wk)	Route				
Wang et al.	HDL-NPs	OV	*Foxn1*^nu^ female mice	1 μM	4	IP	↓	↓	NS ^‡^	[73]
Dash et al.	QAuNPs	BRCA	BALB/c female mice	15 μg/kg	4	Oral	−	↓	↑	[74]
Jiang et al.	Amuc_2172@MMCNPs	CRC	*Apc*^min/+^ mice	150 μg/kg	2	IP	↓	↓	↑	[75]
Ren et al.	PpIX-FFYSV	BRCA	BALB/c female mice	20 mg/kg	2	IV	−	↓	NS	[76]
Abo-Elfadl et al.	PEG-AuNPs	SKCM	CD1 female mice	4 μg/kg	5	IT	−	↓	−	[81]
Kwak et al.	rINN@PEG/PLGA-NPs	CCA	*Foxn1*^nu^ male mice	50 mg/kg (1 mg rINN)	4	SC	−	↓	NS	[83]
Martin et al.	Bel@PGON/PLGA-NPs	BLCA	*Foxn1*^nu^ female mice	5 mg/kg	4	IT	−	↓	−	[84]

↑ and ↓ indicate upregulation and downregulation, respectively. BRCA: breast cancer; BLCA: bladder cancer; CCA: cholangiocarcinoma; CRC: colorectal cancer; IP: intraperitoneal; IT: intratumoral; IV: intravenous; OV: ovarian cancer; SC: subcutaneous; SKCM: skin cutaneous melanoma; * Abbreviations are based on The Cancer Genome Atlas designations. ^†^ Outcome measures comprise tumor count (TC), tumor size (TS) and body weight (BW). ^‡^ NS: not significant.

## Data Availability

The extracted data used in this systematic review are provided as Supplementary File S1.

## Supplementary Materials

The following supporting information can be downloaded at: https://www.mdpi.com/article/10.3390/epigenomes9040044/s1, Checklist S1: Preferred Reporting Items for Systematic reviews and Meta-Analyses extension for Scoping Reviews (PRISMA-ScR) Checklist; Table S1: Risk of bias assessment of in vitro studies based on the ToxRTool checklist; Table S2: Primary keywords used for development of search query strings; Table S3: List of search query strings and results of the systematic search categorized by literature databases. (Updated as of November 2025); File S1 XLSX: Spreadsheet of data extracted from the included studies.

## Abbreviations

The following abbreviations are used in this manuscript:

AgNP	Silver nanoparticle
AsNP	Arsenic oxide nanoparticle
AuNP	Gold nanoparticle
BLCA	Bladder cancer
BRCA	Breast cancer
BSA	Bovine serum albumin
CCA	Cholangiocarcinoma
CDDP	Cisplatin
CRC	Colorectal cancer
DMSA	Dimercaptosuccinic acid
ENT	Entinostat
GON	Graphene oxide nanosheet
HDL	High-density lipoprotein
LUAD	Lung adenocarcinoma
MMCNP	Macrophage membrane-coated nanoparticle
OV	Ovarian cancer
OXP	Oxaliplatin
PEG	Polyethylene glycol
PDAC	Pancreatic ductal adenocarcinoma
PGON	poly(guanidinium oxanorbornene)
PLGA	Poly(lactic-co-glycolic acid)
PpIX	Protoporphyrin IX
PVP	Polyvinylpyrrolidone
PRAD	Prostate adenocarcinoma
rINN	Vorinostat
SKCM	Skin cutaneous melanoma
TSA	Trichostatin A
YSV	Tyroservatide
