# Supplementary material for: Exploring the Impact of Nanotherapeutics on Histone H3 and H4 Acetylation Enrichment in Cancer Epigenome: A Systematic Scoping Synthesis
Source: Epigenomes. 2025 Nov 7;9(4):44. doi: 10.3390/epigenomes9040044 (PMC12641773; doi:10.3390/epigenomes9040044)
Supplement: Supplementary file 1 [file epigenomes-09-00044-s001.zip › Supplementary Tables.pdf]

Table S1. Risk of bias assessment of in vitro studies based on the ToxRTool checklist.

Study	Domain*								Overall Reliability
	1	2	3	4	5	6	7	8	
**Giri et al.**	Yes	Yes	Yes	Yes	Yes	Yes	Yes	Yes	Reliable without restrictions
**Wang et al.**	Yes	Yes	Yes	Yes	Yes	Yes	Yes	No	Reliable with restrictions
**Dash et al.**	Yes	Yes	Yes	Yes	Yes	Yes	Yes	Yes	Reliable without restrictions
**Jiang et al.**	Yes	Yes	No	Yes	No	Yes	Yes	Yes	Reliable with restrictions
**Ren et al.**	Yes	Yes	Yes	Yes	Yes	Yes	Yes	Yes	Reliable without restrictions
**Sima et al.**	Yes	Yes	Yes	Yes	Yes	Yes	Yes	No	Reliable with restrictions
**Surapaneni et al.**	Yes	Yes	Yes	Yes	Yes	Yes	Yes	Yes	Reliable without restrictions
**Lin et al.**	Yes	Yes	No	Yes	Yes	Yes	Yes	Yes	Reliable with restrictions
**Blanco et al.**	Yes	Yes	Yes	Yes	Yes	Yes	Yes	Yes	Reliable without restrictions
**Abo-Elfadl et al.**	No	Yes	Yes	Yes	Yes	Yes	Yes	No	Reliable with restrictions
**Jadhav et al.**	Yes	Yes	No	Yes	Yes	Yes	Yes	No	Reliable with restrictions
**Kwak et al.**	Yes	Yes	Yes	Yes	Yes	Yes	Yes	Yes	Reliable without restrictions
**Martin et al.**	Yes	Yes	Yes	Yes	Yes	Yes	Yes	Yes	Reliable without restrictions

***ToxRTool Domains:**

1. **Test System Identification & Characterization**
2. **Test System Maintenance & Culture Conditions**
3. **Test Substance Identification & Characterization**
4. **Vehicle/Solvent & Its Controls**
5. **Dose Selection & Concentrations**
6. **Exposure Conditions**

7. **Positive/Negative Controls**
8. **Endpoint Measurement & Analytical Methods**

Table S2. Primary keywords used for development of search query strings.

Keyword	Synonym OR Equivalent OR Variant
**Cancer**	Malignancy, neoplasia, neoplasm, tumor, carcinoma, adenocarcinoma, neoplastic disease, neoplastic growth
**Histone acetylation**	Histone acetylase, histone acetyltransferase, histone deacetylase, histone deacetylation, GCN5, P300, EP300, HDAC*, SIRT*, H3K4ac, H3K9ac, H3K27ac, H3K36ac, H3K79ac, H4K5ac, H4K12ac, H4K16, H4K20ac
**Nanoformulation**	Nanomedicine, nanodrug, nanomaterial, nanoparticle, nanocarrier, nanostructured particle, particulate nanostructure, quantum dots, graphene oxide, liposome, dendrimer, nanotube, nanogel

Table S3. List of search query strings and results of the systematic search categorized by literature databases. (Updated as of November, 2025).

Literature Database	Query	String	Records
**Embase**	#1	(cancer:ti,ab,kw OR malignan*:ti,ab,kw OR neoplasia:ti,ab,kw neoplasm:ti,ab,kw OR tumor:ti,ab,kw OR carcinoma:ti,ab,kw OR adenocarcinoma:ti,ab,kw OR 'neoplastic disease':ti,ab,kw OR 'neoplastic growth':ti,ab,kw) OR 'malignant neoplasm'/exp	**5,104,320**
	#2	('histone acetylation':ti,ab,kw OR 'histone acetylase':ti,ab,kw OR 'histone acetyltransferase':ti,ab,kw OR 'histone deacetylase':ti,ab,kw OR 'histone deacetylation':ti,ab,kw OR GCN5:ti,ab,kw OR *P300:ti,ab,kw OR hdac*:ti,ab,kw OR sirt*:ti,ab,kw OR h3k4ac:ti,ab,kw OR h3k9ac:ti,ab,kw OR h3k27ac:ti,ab,kw OR h3k36ac:ti,ab,kw OR h3k79ac:ti,ab,kw OR h4k5ac:ti,ab,kw OR h4k12ac:ti,ab,kw OR h4k16ac:ti,ab,kw OR h4k20ac:ti,ab,kw) OR 'histone acetylation'/exp	**87,556**
	#3	(nanoformulation:ti,ab,kw OR nanomedicine:ti,ab,kw OR nanodrug:ti,ab,kw OR nanomaterial:ti,ab,kw OR nanoparticle:ti,ab,kw OR nanocarrier:ti,ab,kw OR 'nanostructured particle':ti,ab,kw OR 'particulate nanostructure':ti,ab,kw OR 'quantum dots':ti,ab,kw OR 'graphene oxide':ti,ab,kw OR liposome:ti,ab,kw OR dendrimer:ti,ab,kw OR nanotube:ti,ab,kw OR nanogel:ti,ab,kw) OR 'nanoparticle'/exp	**276,881**
	#4	#1 AND #2 AND #3	**97**
**PubMed/MEDLINE**	#1	(cancer[Title/Abstract] OR malignan*[Title/Abstract] OR neoplasia[Title/Abstract] OR neoplasm[Title/Abstract] OR tumor[Title/Abstract] OR carcinoma[Title/Abstract] OR adenocarcinoma[Title/Abstract] OR (neoplastic disease[Title/Abstract]) OR (neoplastic growth[Title/Abstract])) OR "neoplasms"[MeSH Terms] OR "carcinoma"[MeSH Terms] OR "adenocarcinoma"[MeSH Terms]	**5,479,173**
	#2	((histone acetylation[Title/Abstract]) OR (histone acetylase[Title/Abstract]) OR (histone acetyltransferase[Title/Abstract]) OR (histone deacetylase[Title/Abstract]) OR (histone deacetylation[Title/Abstract]) OR HDAC*[Title/Abstract] OR SIRT*[Title/Abstract] OR H3K4ac[Title/Abstract] OR H3K9ac[Title/Abstract] OR H3K27ac[Title/Abstract] OR	**76,076**

		H3K36ac[Title/Abstract] OR H3K79ac[Title/Abstract] OR H4K5ac[Title/Abstract] OR H4K12ac[Title/Abstract] OR H4K20ac[Title/Abstract]) OR (("histones"[Supplementary Concept] OR "histone"[Title/Abstract] OR "histones"[MeSH Terms]) AND "acetylation"[MeSH Terms])	
	#3	nanoformulation[Title/Abstract] OR nanomedicine[Title/Abstract] OR nanodrug[Title/Abstract] OR nanomaterial[Title/Abstract] OR nanoparticle[Title/Abstract] OR nanocarrier[Title/Abstract] OR (nanostructured particle[Title/Abstract]) OR (particulate nanostructure[Title/Abstract]) OR (quantum dots[Title/Abstract]) OR (graphene oxide[Title/Abstract]) OR liposome[Title/Abstract] OR dendrimer[Title/Abstract] OR nanotube[Title/Abstract] OR nanogel[Title/Abstract] OR "nanoparticles"[MeSH Terms] OR "nanomedicine"[MeSH Terms] OR "liposomes"[MeSH Terms] OR "dendrimers"[MeSH Terms] OR "nanogels"[MeSH Terms]	**425,560**
	#4	#1 AND #2 AND #3	**243**
**Scopus**	#1	TITLE-ABS-KEY (cancer) OR TITLE-ABS-KEY (malignan*) OR TITLE-ABS-KEY (nroplasia) OR TITLE-ABS-KEY (neoplasm) OR TITLE-ABS-KEY (tumor) OR TITLE-ABS-KEY (carcinoma) OR TITLE-ABS-KEY (adenocarcinoma) OR TITLE-ABS-KEY (neoplastic disease) OR TITLE-ABS-KEY (neoplastic growth)	**6,969,916**
	#2	TITLE-ABS-KEY (histone acetylation) OR TITLE-ABS-KEY (histone acetylase) OR TITLE-ABS-KEY (histone acetyltransferase) OR TITLE-ABS-KEY (histone deacetylase) OR TITLE-ABS-KEY (histone deacetylation) OR TITLE-ABS-KEY (GCN5) OR TITLE-ABS-KEY (*P300) OR TITLE-ABS-KEY (HDAC*) OR TITLE-ABS-KEY (SIRT*) OR TITLE-ABS-KEY (H3K4ac) OR TITLE-ABS-KEY (H3K9ac) OR TITLE-ABS-KEY (H3K27ac) OR TITLE-ABS-KEY (H3K36ac) OR TITLE-ABS-KEY (H3K79ac) OR TITLE-ABS-KEY (H4K5ac) OR TITLE-ABS-KEY (H4K12ac) OR TITLE-ABS-KEY (H4K16ac) OR TITLE-ABS-KEY (H4K20ac)	**135,961**
	#3	TITLE-ABS-KEY (nanomedicine) OR TITLE-ABS-KEY (nanodrug) OR TITLE-ABS-KEY (nanomaterial) OR TITLE-ABS-KEY (nanoparticle) OR TITLE-ABS-KEY (nanocarrier) OR TITLE-ABS-KEY (nanostructured particle) OR TITLE-ABS-KEY (particulate nanostructure) TITLE-ABS-KEY (quantum dots) OR TITLE-ABS-KEY (graphene oxide)	**219,471**

		OR (liposome) OR TITLE-ABS-KEY (dendrimer) OR TITLE-ABS-KEY (nanotube) OR TITLE-ABS-KEY (nanogel)	
	#4	#1 AND #2 AND #3	**248**
**Web of Science**	#1	TS=(cancer) OR TS=(malignan*) OR TS=(neoplasia) OR TS=(neoplasm) OR TS=(tumor) OR TS=(adenocarcinoma) OR TS=(neoplastic disease) OR TS=(neoplastic growth)	**4,490,792**
	#2	TS=(histone acetylation) OR TS=(histone acetylase) OR TS=(histone acetyltransferase) OR TS=(histone deacetylase) OR TS=(histone deacetylation) OR TS=(GCN5) OR TS=(*P300) OR TS=(HDAC*) OR TS=(SIRT*) OR TS=(H3K4ac) OR TS=(H3K9ac) OR TS=(H3K27ac) OR TS=(H3K36ac) OR TS=(H3K79ac) OR TS=(H4K5ac) OR TS=(H4K12ac) OR TS=(H4K16ac) OR TS=(H4K20ac)	**121,867**
	#3	TS=(nanomedicine) OR TS=(nanodrug) OR TS=(nanomaterial) OR TS=(nanoparticle) OR TS=(nanocarrier) OR TS=(nanostructured particle) OR TS=(particulate structure) OR TS=(quantum dots) OR TS=(graphene oxide) OR TS=(liposome) OR TS=(dendrimer) OR TS=(nanotube) OR TS=(nanogel)	**1,026,936**
	#4	#1 AND #2 AND #3	**245**
Total			**833**
